# Developing proxy indicators of healthcare associated infections to support syndromic surveillance in a UK academic health science centre

**DOI:** 10.1186/1753-6561-5-S6-P33

**Published:** 2011-06-29

**Authors:** C King, L Garcia Alvarez, A Holmes, P Aylin

**Affiliations:** 1 Centre for Infection Prevention and Management, Imperial College London, London, UK

## Introduction / objectives

A wealth of disparate data are routinely collected and stored within public hospitals in England, providing a potential source of information for novel surveillance tools for healthcare associated infections (HCAIs). In the absence of a complete electronic patient record, this study aims to utilise these data to predict HCAIs and enhance early identification.

## Methods

A systematic literature review was employed to develop an evidence-based inventory of risk factors for HCA *Clostridium difficile*, surgical site infections, urinary tract infections, bloodstream infections and pneumonia.

Data from multiple internal hospital databases were linked, using an encoded patient ID, into a coherent database with information on demographics, admissions, diagnostics, procedures and microbiology. The database was interrogated for the presence of a range of risk factors identified by the literature review, to develop predictive risk models and proxy infection indicators. Cases of HCAIs were extracted from the database for the purpose of risk modelling.

## Results

The literature review resulted in 341 papers, providing 293 independent risk factors of HCAIs.

The database contains 370,559 administrative inpatient care records from 2007-10, corresponding to 310,722 microbiology tests, 628,861 diagnostic codes and 367,550 procedure codes. So far, 47 risk factors have been identified and predictive risk models and proxy indicators are being developed to support syndromic surveillance.

## Conclusion

Routinely collected hospital data can be used to identify risk factors for HCAIs and develop proxy indicators of infection and risk. This resource has potential for innovative surveillance tools that could be implemented in real time and impact on the incidence of HCAIs within acute care settings.

## Disclosure of interest

None declared.

